# Retracted: Design of Financial Risk Control Model Based on Deep Learning Neural Network

**DOI:** 10.1155/2023/9780975

**Published:** 2023-03-01

**Authors:** Computational Intelligence and Neuroscience

*Computational Intelligence and Neuroscience* has retracted the article titled “Design of Financial Risk Control Model Based on Deep Learning Neural Network” [1] due to concerns that the peer review process has been compromised.

Following an investigation conducted by the Hindawi Research Integrity team [2], significant concerns were identified with the peer reviewers assigned to this article; the investigation has concluded that the peer review process was compromised. We therefore can no longer trust the peer review process, and the article is being retracted with the agreement of the Chief Editor.

The authors agree to the retraction.

## References

[1] Yang D., Ma H., Chen X., Liu L., Lang Y. (2022). Design of Financial Risk Control Model Based on Deep Learning Neural Network. *Computational Intelligence and Neuroscience*.

[2] Ferguson L. (2022). Advancing Research Integrity Collaboratively and with Vigour. https://www.hindawi.com/post/advancing-research-integrity-collaboratively-and-vigour/.

